# Simplifying Serious Illness Communication: Preparing or Deciding

**DOI:** 10.3390/curroncol31100433

**Published:** 2024-09-28

**Authors:** Jeff Myers, Leah Steinberg, Nadia Incardona, Jessica Simon, Justin Sanders, Hsien Seow

**Affiliations:** 1 Department of Family and Community Medicine, University of Toronto, Toronto, ON M5S 1A1, Canada; leah.steinberg@sinaihealth.ca (L.S.); nadia.incardona@utoronto.ca (N.I.); 2 Department of Oncology, Medicine and Community Health Sciences, University of Calgary, Calgary, AB T2N 1N4, Canada; jessica.simon@albertahealthservices.ca; 3 Department of Family Medicine, Université McGill, Montreal, QC H3A 0G4, Canada; justin.sanders@mcgill.ca; 4 Department of Oncology, McMaster University, Hamilton, ON L8S 4L8, Canada; seowh@mcmaster.ca

**Keywords:** serious illness communication, advance care planning, goals-of-care discussions, serious illnesses, communication skills

## Abstract

Clinicians have a sincere desire to ensure that the decision-making processes of seriously ill patients are well informed throughout illness trajectories. A quagmire of variable terminology (e.g., advance care planning, serious illness conversations, goals-of-care discussions, etc.), however, currently predominates the field of serious illness communication. This creates uncertainty among clinicians as to the overall purpose, tasks, and specific outcomes of conversations that address serious illness. The *Preparing or Deciding* model is a unifying framework that provides conceptual clarity by helping clinicians understand their role in leading these important conversations. The *Preparing or Deciding* model simply posits that conversations with seriously ill patients are about either preparing or deciding. It considers these tasks to be mutually exclusive, which can help bypass many of the barriers to having these conversations. The *Preparing or Deciding* model compliments all existing resources and frameworks and is applicable to all healthcare practitioners in every care setting. To help move forward serious illness communication education and research, as well as process improvement efforts more effectively, here, we describe the *Preparing or Deciding* model.

High-quality discussions that address serious illness are more likely to meet the psychosocial, emotional, and information needs of seriously ill patients and their families, as well as improve outcomes at the individual and population levels [1,2]. When directly interacting with patients, however, many health care practitioners (i.e., clinicians) find themselves caught in the quagmire of variable terminology that currently predominates the emerging field of serious illness communication [3]. Advance care planning (ACP), for example, has recently been the subject of much debate regarding its essential components and efficacy [4,5]. Defined by an international panel of experts as “a process for a person of any age or stage of health to understand and share personal values, life goals, and preferences regarding future medical care” [6], ACP, in theory, links a person’s values and goals with the delivery of better and more goal-concordant care. Decades of mixed research findings, however, have led to controversy around its value.

Despite a sincere desire to support decision-making throughout the course of serious illnesses, most clinicians are uncertain about their role in serious illness communication. This uncertainty, combined with complex multi-morbidity and competing priorities in clinical encounters, confounds the well-intended efforts to encourage clinicians to have supportive, proactive conversations with their seriously ill patients. There is a pressing need to provide clear guidance to clinicians about how to engage patients in high value conversations throughout serious illnesses.

## 1. Case

James, an 81-year-old, has recently relapsed diffuse B-cell lymphoma and NYHA Class II heart failure from the cardiotoxic effects of previous treatments. James recently learned that his treatment options are limited and that he is unlikely to be offered CAR T-cell therapy. His heart failure, along with grade 4 chronic kidney disease, led to two hospitalizations this year. Kathryn, James’ spouse and substitute decision maker (SDM), regularly attends his appointments, including the most recent post-admission follow up. The clinician seeing James understands that he is living with serious, progressive illnesses and that it is an important time to discuss what this means. However, the clinician is unsure how to prioritize this among the many post-admission tasks, as well as what specifically should be addressed. How should the discussion be introduced? Is ACP or a serious illness conversation needed? How do these differ from a goals-of-care discussion (GOCD)? What is James’ prognosis? Should code status be discussed? How should James’ preferences for care in the future be approached?

## 2. The Problem

The clinician’s challenges in this scenario include the perception of not having adequate prognostic information and not wanting to take hope away from James and Kathryn. The most problematic challenge, however, is uncertainty about which conversations related to serious illness take precedent. Conflicting conceptual models for serious illness communication and an alphabet soup of acronyms to describe the related tasks further undermine clinician confidence in leading these important conversations. The need for a framework that can help clinicians navigate these persistent challenges and return focus to patients is urgent.

## 3. A Solution—The Preparing or Deciding (POD) Model

**Figure 1:**
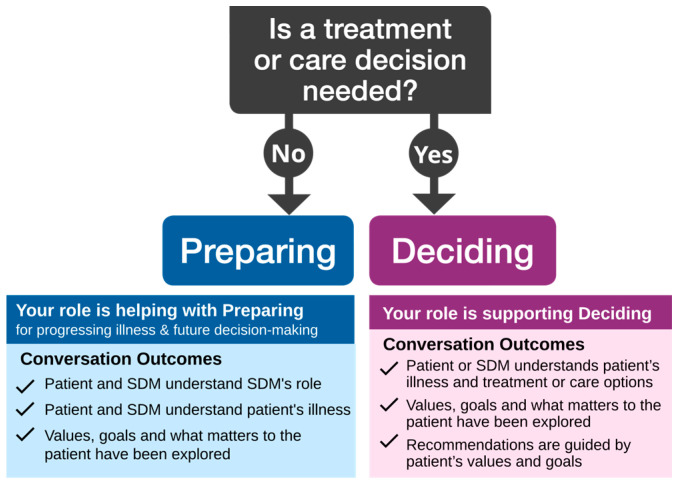
The *Preparing or Deciding* model.

We propose the POD model (Figure 1), a unifying conceptual framework to help clinicians understand their role in serious illness communication by clarifying the overall purpose, tasks, and specific outcomes of conversations. It posits that conversations with seriously ill patients are, at a high level, about either preparing or deciding. In practice, during any interaction with a person affected by serious illness, a clinician asks themselves: *Is a decision needed now?* If yes, they need conversational approaches that support decision-making processes. If no, their focus is on helping the patient or their SDMs prepare for what lies ahead, including decisions that might arise. From this single question, the model guides clinicians on the specific tasks of preparing or deciding. 

**Figure 2:**
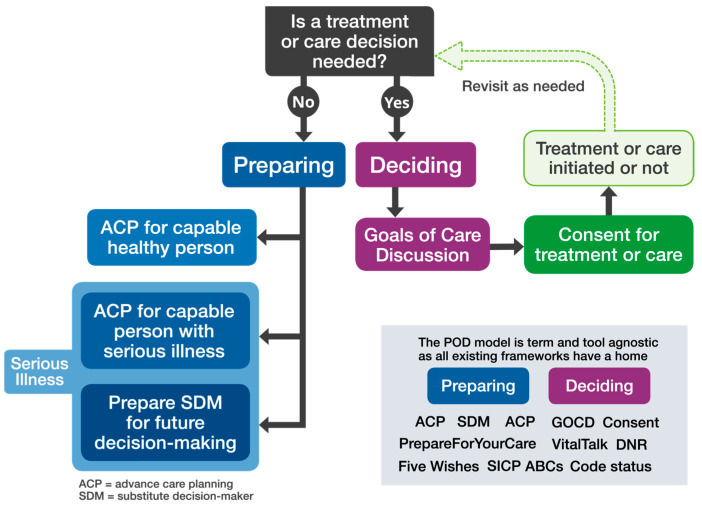
Expanded *Preparing or Deciding* model.

Others have attempted to resolve the confusion surrounding ACP and GOCD by situating each on timelines that suggest the appropriateness of one or the other based on the stage of life or illness [7] or by deriving consensus definitions [6]. The iterative nature of these conversations, however, makes timeline-based conversations challenging [8]. The POD model helps to address this confusion by directly linking ACP and GOCD to the core task associated with each: preparing and deciding (Figure 2).

Over the last 10–15 years, substantial effort and resource have been aimed at the evolving field of serious illness communication. This has included the development of several resources, programs, and quality improvement interventions (e.g., Serious Illness Care Program, Five Wishes, VitalTalks, Prepare for your care) [2,9,10,11]. Using preparing or deciding to clarify the purpose of conversations compliments these efforts and provides additional structure to support those who are advancing their communication skills. (Figure 2).

Considering the tasks of preparing and deciding as mutually exclusive addresses the recent critiques of ACP by avoiding the common pitfall of using ACP to make decisions about interventions that may or may not be offered in the future, e.g., dialysis, resuscitation, and tube feeds. A person cannot make treatment or care decisions in the absence of clinical context and this treatment-centred approach to ACP is known to be ineffective, i.e., advance directives and living wills frequently fail to guide decision-making or improve the delivery of goal-consistent care [12]. The POD model offers a clear heuristic to help clinicians support decision-making when appropriate, and to otherwise understand that conversations addressing serious illness are to prepare.

Despite differences across health systems in documentation standards and medical and legal frameworks that support decision-making and serious illness communication, the POD model is applicable in every care setting and to all healthcare practitioners. The task of preparing can also include “healthy, capable adults” (Figure 2). Of particular benefit to researchers and those involved in quality improvement, the model also clarifies conversation outcomes (Figure 1).

## 4. What Is Involved with Preparing?

Preparing a seriously ill person and their SDM involves them both understanding the SDM role, assessing and addressing illness understanding, and exploring the person’s values and goals. Preparing should be thought of as a longitudinal and iterative process for times when decisions are not needed. In practice, this means engaging patients and SDMs in multiple conversations over time.

Because most seriously ill people have unmet information needs, addressing illness understanding is essential when preparing [13,14,15]. This requires clarifying information preferences, as well as providing information about illness trajectories. Clinicians often view this task as communicating prognosis or prognostic disclosure and commonly take prognosis to mean expected survival duration. The inherent uncertainty with survival estimates creates a barrier to engaging in preparing conversations, as the task of prognostication is often misconceived as one that necessitates precision, rather than as one intended to help people prepare for the possibility of dying or further disability [16]. The focus of prognosis should be on the components for which there is more certainty, such as illness being incurable, that progressive functional decline is expected, that exacerbations are likely to be recurrent, and that there is ongoing potential for sudden decline. 

Using the above case as an example, James and Kathryn would be more prepared if they understood (1) that neither the lymphoma or the heart failure can be cured, (2) that future exacerbations are expected, and (3) that James’ function is expected to decline over time. Revisiting these understandings in between decisions may improve decision-making processes overall. 

Throughout the trajectory of serious illness, preparing can occur in any setting and should not be thought of as a primary or specialty care task or as specific to any profession. For example, in this case, preparing conversations could occur with James during the ambulatory care follow-up visit described in the scenario and could also have occurred in hospital while he was still an inpatient. The proximity of clinical context from James’ hospital experience provides an opportunity for preparing, which could include asking James and Kathryn: What do you understand about why James needed to be in hospital? How was it for him to be there? Would it be a surprise if this were to happen again? Listening carefully to their answers can lead to the discovery of unmet information needs.

## 5. What Is Involved with Deciding?

The initial tasks of deciding are similar to those for preparing, i.e., assessing and addressing illness understanding, as well as exploring values and goals. Key differences when deciding are found in the roles for both clinical context and values and goals. While clinical context can be helpful when preparing, it is essential to deciding. Only when clinical context is fully appreciated can a person contemplate what matters, formulate goals, and make decisions that are both informed and shared [17].

Like clinical context, while values and goals are helpful to reflect on when preparing, they are directly relevant to deciding. Information about what matters to a person, what they imagine specific treatments will allow them to achieve, and what worries they have about the future is critical to effective decision-making. In current practice, however, as few as 10% of clinician recommendations are guided by a patient’s values and goals [18]. A clinician who keeps the person at the centre of decision-making bases their recommendations about specific treatment or care plan options on information about a person’s values and goals.

Clinicians often view a person’s goals that are incongruent or incompatible with the clinical picture as problematic. They may feel obliged to provide corrective information or they may simply discount a goal as not being possible. However, clinicians should seek to understand why the goal is important to the person. This may uncover an information gap or difficulties with coping. If there is uncertainty around the likelihood that a treatment will achieve a person’s goal, trials can be pursued, with the person’s goals used as end points for determining success [19]. 

Among studies exploring the timing of decision-making discussions in people with serious illness, it is commonly recommended that these occur earlier in illness trajectories [20]. Maintaining focus in-between decisions on adequately preparing a person or SDM for progressing illness may avoid the problems that emerge when it is presumed decisions can be made prior to treatments or care being offered.

## 6. Conclusions

The POD model aims to provide conceptual clarity such that clinicians feel less stuck or bogged down by terminology when navigating conversations about serious illness. Recognizing that conversations are either for preparing or deciding can help bypass many of the barriers to having these conversations. When clinicians better understand their role in serious illness communication, individuals, teams, and systems can more effectively move forward with education and research and process improvements.
